# Impact of Depression on Patients With Hemophilia: A Retrospective Case-Control Research

**DOI:** 10.3389/fpsyt.2022.892321

**Published:** 2022-07-04

**Authors:** Ana María Jiménez-Cebrián, Patricia Palomo-López, Ricardo Becerro-de-Bengoa Vallejo, Marta Elena Losa-Iglesias, Emmanuel Navarro-Flores, Marta San-Antolín, César Calvo-Lobo, Daniel López-López

**Affiliations:** ^1^Department Nursing and Podiatry, Faculty of Health Sciences, Instituto de Investigación Biomédica de Málaga (IBIMA), University of Málaga, Málaga, Spain; ^2^University Center of Plasencia, University of Extremadura, Plasencia, Spain; ^3^Facultad de Enfermería, Fisioterapia y Podología, Universidad Complutense de Madrid, Madrid, Spain; ^4^Faculty of Health Sciences, Universidad Rey Juan Carlos, Alcorcón, Spain; ^5^Frailty Research Organized Group (FROG), Department of Nursing, Faculty of Nursing and Podiatry, University of Valencia, Valencia, Spain; ^6^Department of Psychology, Universidad Europea de Madrid, Madrid, Spain; ^7^Research, Health and Podiatry Group, Department of Health Sciences, Faculty of Nursing and Podiatry, Industrial Campus of Ferrol, Universidade da Coruña, Ferrol, Spain

**Keywords:** Beck Depression Inventory, depression, foot health, hemophilia, healthy

## Abstract

**Background:**

Hemophilia is an inherited recessive hemorrhagic disorder of the X-linked type, suffered by the male sex. Adults with hemophilia are coping with numerous diagnostics, associated comorbidities, pain, and difficult gait by arthropathy in ankles and feet. Physical pains contribute to depression in patients with hemophilia.

**Purpose:**

The study aimed to assess the impact of depression in adult patient with hemophilia and to compare it with healthy matched-paired controls. *Methods:* The sample consisted of 100 participants (median age 42.50 ± 30). Patients with hemophilia were recruited from Spanish Hemophiliac Associations (*n* = 50) and healthy subjects (*n* = 50) from a Clinic Podiatry Practices (University of Malaga, Spain).

**Results:**

Results and categories of the Spanish-translated version of the Beck Depression Inventory (BDI) were gathered. A clear statistically significant difference (*p* < 0.001) was presented in the variation of the BDI scores between both groups. Patients with hemophilia presented worse results with a BDI = 7.50 ± 11.25 points compared to healthy subjects with BDI = 2.50 ± 5 points. In the BDI categories, statistically significant differences (*p* = 0.004) were found in greater BDI categories in the Hemophilia group compared with healthy subjects. Moderate and severe depression categories were only shown in patients with hemophilia.

**Conclusions:**

Greater depression scores and range status were observed in patients with hemophilia compared to non-patients with hemophilia. Patients with hemophilia are at increased risk of depressiveness.

## Introduction

Hemophilia is an inherited recessive hemorrhagic disorder of the X-linked type, suffered by the male sex. There are principally 2 forms of Hemophilia: Hemophilia A, the most usual type caused by a missing clotting factor VIII (prevalence 1 case per 10,000 men), whereas Hemophilia B is produced by the absence of factor IX (prevalence 1 patient every 30,000 men) (1, 2).

A normal and recurrent manifestation of serious Hemophilia A and B is non-traumatic (spontaneous) intra-articular hemorrhage (i.e., hemarthrosis). The most frequent site of hemorrhage is the ankle, succeeded by the elbow and the knee (3). Frequent hemarthrosis may cause progressive joint harm, shown by synovial hypertrophy and osteochondral injury. Progressively, the development of hemophilic arthropathy causes severe functional limitations (4) and painful joint deformations (5).

This chronic illness is avowed as a significant public health problem, due to the negative impact posing for these patients, your families and health and/or economic burden, with consequent available resources, financial, medical practice, and socioeconomic implications affecting the healthcare (6, 7).

On the order hand, various investigations have indicated the existence of a relationship between psychological disorders and different musculoskeletal variables (i.e., progress, prognostication, repetition, and gravity of outbreak), with depression and anxiety being regularly implicated as emotional factors in the context (8–10).

However, at present, there are insufficient studies in Spain that examine the seriousness of depression taking into account its affective, behavioral, and cognitive dimensions and anxiety in patients with hemophilia. In this context, hemophilia is one general pathology in which older patients with hemophilia are frequently faced with numerous diagnostics, associated comorbidities, and pain due to arthropathy affectations (9).

Despite the importance of this chronic disease, both due to its prevalence and its influence on the daily activities of living, there are insufficient studies in Spain that have measured the influence of anxiety and depression in these patients. The study aimed to assess the impact of depression in adult patients with hemophilia and also to compare healthy subjects with healthy matched-paired controls.

## Experimental Section

### Design and Sample

We performed an observational, cross-sectional, descriptive, and case-control research in agreement with Strengthening the Reporting of Observational Studies in Epidemiology Statement (STROBE) guidelines. Using a consecutive sampling method, a total sample of 100 subjects was selected from the Hemophiliac Associations of Málaga, Aragon, and Cantabria (Spain) and a University Clinic (University of Málaga, Spain) between December 2019 and February 2020. Eligible participants were male subjects with hemophilia type A or B diagnosed by a hematologist (case group, *n* = 50) and male salubrious subjects (control group, *n* = 50). The participant selection and inclusion were: (1) male gender (2) older than 18, (3) persons with Hemophilia diagnosed by a specialist physician and not having neurological or psychological problems (case group), and persons without hemophilia and health problems (control group). The exclusion rule were: (1) people with depression, (2) antecedents of previous hematologic disease, (3) neurological problems, (4) a history of surgery and/or orthopedic problems, (5) loss of independence or self-sufficiency in daily routines, (6) refusal to approve the consent form, and (7) cognitive impairment of any etiology.

### Procedure

Baseline measures included general questions associated with (1) demographic variables (age, weight, height, job status, level of studies, and marital status); (2) characteristics of comorbid problems (hyperglycemia, overweight, musculoskeletal difficulties, and circulatory disorders); (3) foot problems. In addition, exact health questions associated with hemophilia, such as (4) type and (5) clinical grade of hemophilia, were recorded. Next, participants completed the BDI questionnaire (11). This interrogation was interpreted to Spanish and evidenced to become a tested instrument used for assessment of depression that was constituted with 21 questionings, each question had on a scale between 0 and 3 points, giving a total result between 0 and 63 points. The result analysis was registered about the next classification: (1) from 0 to 9:no sign of depression, (2) from 10 to 15: mild depression, (3) from 16 to 23: borderline depression, and (4) from 24 to 57: serious depression. This instrument could be considered a simple and exact exam for the evaluation of persons with indications of depression (12). BDI has an internal consistency that demonstrates a Cronbach's alpha coefficient of 0.889 (13).

### Ethical Considerations

This study got a positive report issued by the Ethical Committee from the University of Extremadura (Spain, registry code: 263/2019). Ethical principles for medical research on humans to the Helsinki Declaration (World Medical Association) were respected (14). The informed consent form was signed by all participants before being included in the investigation.

### Sample Size Calculation

The G ^*^ Power software (version 3.1.9.2, Universität Düsseldorf, Düsseldorf, Germany) was used to calculate the sample size, using the differences between 2 groups of independent samples, based on a preliminary investigation (*n* = 32) with 2 groups (mean ± SD) and on the scores of the Beck Depression Inventory (BDI), 16 patients with hemophilia (case group, BDI = 6.50 ± 5.50 points) and 16 sane paired participants (control group, BDI = 3.12 ± 2.57 points). Also, for sample size calculation were used: 2-tailed test, the effect size of 0.78, the alpha error probability of 0.01, the power (probability of 1-beta error) of 0.90, and 1 allocation ratio (N2/N1). Therefore, a final sample size of 100 subjects, 50 persons with Hemophilia and 50 healthy matched-paired persons, was resolved with an actual power of 0.902.

### Statistical Analysis

Statistical analyses were performed using the SPSS 24.0 (IBM Corp., Armonk, NY, USA). For all analyses, Statistical significance was established at *P* < 0.05 and a 95% confidence interval (CI).

Regarding the quantitative variable, the normality was evaluated by the Kolmogorov-Smirnov test. All data were distributed as non-parametric data (Kolmogorov–Smirnov test showed a *p*-value minor than 0.05) and median and interquartile range (IR), as well as maximum and minimum (range) values, were utilized to describe the total sample. Mann–Whitney *U*-tests were used to obtain if the differences between both groups were statistically significant.

Concerning categorical variables, frequencies and percentages were applied. The Chi-square test was used to assess differences between both groups (BDI category).

## Results

### Descriptive Data

A sample of 100 male subjects finished the research and was classified into two groups, patients with hemophilia and persons without hemophilia (case group, *n* = 50), and salubrious matched-paired participants (control group, *n* = 50) presented an age division from 18 to 80 years old. Furthermore, the participants with hemophilia revealed two types of this chronic illness, type A (90%) showed clinical grades of mild (18%), moderate (14%), and serious (68%), while type B is at 10%. Also, the patients with hemophilia stated they had suffered foot pain (86%) and ankle arthropathy (76%). Statistically significant differences were not shown (*p* > 0.01) between the two groups for descriptive data (Table 1).

**Table 1:** Descriptive data of the patients with hemophilia and healthy matched-paired controls.

**Descriptive Data**	**Total Group** **(*n* = 100)**	**Hemophilic** **(*n* = 50)**	**Healthy** **(*n* = 50)**	***p*-Value**
Age (years)	42.50 ± 30.00 (18–80)	42.50 ± 30.75 (18–80)	42.50 ± 30.75 (18–80)	1.000†
Weight (kg)	77.00 ± 17.50 (56–140)	76.00 ± 19.00 (56–140)	78.00 ± 15.75 (58–100)	0.994†
Height (m)	1.76 ± 0.10 (1.55–1.90)	1.76 ± 0.10 (1.56–1.85)	1.76 ± 0.10 (1.55–1.90)	0.972†
BMI (kg/m^2^)	25.20 ± 4.15 (19.30–40.90)	25.45 ± 4.40 (19.30–40.90)	25.20 ± 4.05 (20.20–33.20)	1.000†

*BMI, body mass index. †, median ± interquartile range, range (min–max), and Mann–Whitney U-test were used*.

*In all the analyses, p < 0.05 (with a 95% confidence interval) was considered statistically significant*.

### Outcome Measurements

A clear statistically significant difference (*p* < 0.005) was presented for the division of the BDI punctuations between both groups (Table 2). Regarding results, subjects who suffered from hemophilia presented worse results of the BDI = 7.50 ± 11.25 points (higher BDI scores) compared to healthy subjects with BDI = 2.50 ± 5 points (lower BDI results). Regarding the BDI categories, there were statistically significant differences (*p* = 0.004) in greater BDI categories in a hemophilic group compared with healthy subjects. It is worthy to note that moderate and severe depression categories were only shown in patients with hemophilia (Table 2).

**Table 2:** Comparisons of BDI scores and categories among patients with hemophilia and healthy matched-paired controls.

**Outcome Measurements**	**Total Group** **(*n* = 100)**	**Hemophiliac** **(*n* = 50)**	**Healthy** **(*n* = 50)**	***p*-Value** **(Hemophiliac vs. Healthy)**	
BDI Category*	No Depression	82 (82%)	34 (68%)	48 (98%)	0.004*
	Mild	14 (14%)	12 (24%)	2 (2%)	
	Moderate	3 (3%)	3 (6%)	0 (0%)	
	Severe	1 (1%)	1 (2%)	0 (0%)	
BDI scores		4.00 ± 6.00 (0.00–32.00)	7.50 ± 11.25 (0.00–32.00)	2.50 ± 5.00 (0.00–16.00)	**<0.001**†

*BDI, Beck depression inventory. Frequency, percentage (%) and Chi-squared test (χ^2^) were utilized. BDI domains were divided as: (1) 0 to 9 points without depression, (2) 10 to 15 points: mild depression, (3) 16 to 23 points: moderate depression, (4) 24 to 57 points: severe depression. †BDI scores, Median ± interquartile range, range (min–max) and Mann–Whitney U test were used. In all the analyses, p < 0.01 (with a 99% confidence interval) was considered statistically significant (**bold**).

## Discussion

The current investigation was performed to contrast the impact of depression in adult patients with hemophilia also to compare with healthy subjects with healthy matched-paired controls.

To the researchers' knowledge, there are insufficient studies in Spain that have measured the influence of anxiety and depression in these patients related with the foot health.

Foot and ankle health is vital to patients with hemophilia, largely due to the major prevalence of musculoskeletal pain in different anatomical areas. Foot and ankle problems have been identified as a public health threat as stated by Wilkins et al., who selected a sample by for high compliance with prophylaxis and analyses of 2.238 participants with hemophilia, reporting severe type A (1889) and type B (349), where one-third of children and two-thirds of adults were shown to have musculoskeletal bleeding, in this anatomical region (15).

To the researchers' knowledge, this study may be considered one of the few investigations in patients with hemophilia using the BDI questionnaire (16) to measure levels of depression. Previous a research had been undertaken to assess the effect on foot wellness of the allergic asthma associated to quality of life and depression using this questionnaire (10). Previously, a research had studied the perception of quality of life linked with general health and depression in pediatric populations of patients with hemophilia (17), and Kim and collaborators studied quality of life and depression in Korean adults with hemophilia using the BDI questionnaire (18).

The results of our study confirm that people with hemophilia presented a “mild depression status” (24%), contrasted to healthy group without hemophilia (2%). Consequently, this research establishes that in patients with hemophilia, exist a relation with BDI depression results. Instead, some studies associate pain in different musculoskeletal conditions with worse physical and psychological factors (9, 19–21). In future studies, factors should also be analyzed regarding the risk that can trigger a depression, one of them may be chronic pain. Chronic pain resulting from joint damage is common in hemophilia. Pain in chronic health states is frequently related to depression (22). In the research of Siboni et al. (23), two-thirds of patients with hemophilia suffered moderate pain/discomfort contrasted to a quarter of people without hemophilia. Physical problems in limbs and difficulties in walking can contribute to the depression. There is evidence that depression is frequent in the elderly population (24–26). In our findings, median age of the subjects were 42.50 years and older patients with hemophilia were 80 years.

Furthermore, it should be taken into account in patients with hemophilia that probability of depression is increased by the factor of suffering from chronic disease and chronic joint pain (27, 28). Thus, certainly, suffering persons from general and hereditary diseases have reaction from an emotional viewpoint (2).

In addition to this, we found that hemophiliac majority subjects could suffer from overweight, being the median BMI was 25.45 in patients with hemophilia group. According to Wilding et al., the incidence of overweight and obesity in persons with hemophilia in Europe and USA is presently comparable to the universal people at 31%, and there has been an important increment in pediatric hemophilic population (29). In entire population, obesity is linked with a raised possibility of high arterial tension, type two diabetes, collapse, coronary heart disease, bone disease, and clinical depression. In patients with hemophilia, obesity has also been connected with limited range of movement in articulations and incremented persistent pain (29, 30). The increased incidence of overweight and obesity in patients with hemophilia notably increments the probability of extending extra comorbidities with aging (28).

Contrasting our results with implications of other studies was not possible due to the contrast in methodological and criteria variations.

Further, this investigation had some limitations. First, a future study with bigger sample sizes, as well as longitudinal evaluation of results, would facilitate in determining definitive conclusions. Second, though appropriate validity and reliability have been created for the BDI questionnaire, their reliability has not yet been recognized expressly for patients with hemophilia and should be considered for future studies. Other limitation of the present study is heterogeneous characteristic s of the sample (subjects who lives in different locations of Spain). Ultimately, it would become of advantage to study patients with hemophilia and with depression in terms of factually calculated results, for example, a clinical and radiological evaluations of articulation state.

## Conclusions

Greater depression scores and ranges status was observed in patients with hemophilia compared to patients without hemophilia. Patients with hemophilia are at increased risk of depressiveness. Thus, individuals from this group should be monitored for potential depressive symptoms. Because hemophilia is a rare disease that requires a multidisciplinary approach, to promote physical and psychological wellness, patients should be attended in comprehensive care of hemophilia treatment center, including psychological evaluation and care.

## Data Availability Statement

The raw data supporting the conclusions of this article will be made available by the authors, without undue reservation.

## Ethics Statement

The studies involving human participants were reviewed and approved by University of Extremadura (Spain, registry code: 263/2019). The patients/participants provided their written informed consent to participate in this study.

## Author Contributions

AJ-C, PP-L, RBV, ML-I, EN-F, MS-A, CC-L, and DL-L: conceptualization, formal analysis, methodology, supervision, writing—original draft, and writing—review and editing. AJ-C: data curation. All authors contributed to the article and approved the submitted version.

## Conflict of Interest

The authors declare that the research was conducted in the absence of any commercial or financial relationships that could be construed as a potential conflict of interest.

## Publisher's Note

All claims expressed in this article are solely those of the authors and do not necessarily represent those of their affiliated organizations, or those of the publisher, the editors and the reviewers. Any product that may be evaluated in this article, or claim that may be made by its manufacturer, is not guaranteed or endorsed by the publisher.

## References

[1] Peyvandi F Garagiola I Young G. The past and future of Hemophilia: diagnosis, treatments, and its complications. Lancet. (2016) 388:187–97. 10.1016/S0140-6736(15)01123-X 26897598

[2] Fiorillo L De Stefano R Cervino G Crimi S Bianchi A Campagna P. Oral and psychological alterations in haemophiliac patients. Biomedicines. (2019) 7:33. 10.3390/biomedicines7020033 31010003 PMC6631232

[3] Stephensen D Tait R Brodie N Collins P Cheal R Keeling D. Changing patterns of bleeding in patients with severe Hemophilia A. Hemophilia. (2009) 15:1210–4. 10.1111/j.1365-2516.2008.01876.x 19659938

[4] Lobet S McCarthy A Hermans C Peerlinck K Matricali GA Staes F. Biomechanical markers and theoretical concepts related to haemophilic ankle and subtalar joint arthropathy: introducing the term “haemophilic tarsal pan-arthropathy”. Haemophilia. (2017) 23:e250–8. 10.1111/hae.13202 28306191

[5] Fijnvandraat K Cnossen MH Leebeek FWG Peters M. Diagnosis and management of Hemophilia. BMJ. (2012) 344:e2707. 10.1136/bmj.e2707 22551712

[6] Chen CX Baker JR Nichol MB. Economic Burden of Illness among Persons with Hemophilia B from HUGS Vb: Examining the Association of Severity and Treatment Regimens with Costs and Annual Bleed Rates. Value Heal. (2017) 20:1074–82. 10.1016/j.jval.2017.04.017 28964439

[7] Nerich V Tissot E Faradji A Demesmay K Bertrand MA Lorenzini JL. Cost-of-illness study of severe Hemophilia A and B in five French Hemophilia treatment centres. Pharm World Sci. (2008) 30:287–92. 10.1007/s11096-007-9181-4 18085428

[8] Patten SB Adair CE Williams JVA Brant R Jian LW Casebeer A. Assessment of mental health and illness by telephone survey: experience with an Alberta mental health survey. Chronic Dis Can. (2006) 27:99–109. 17306061

[9] López DL Fernández JMV Iglesias MEL Castro CÁ Lobo CC Galván JR. Influence of depression in a sample of people with hallux valgus. Int J Ment Health Nurs. (2016) 25:574–8. 10.1111/inm.12196 26892262

[10] López-López D Painceira-Villar R García-Paz V Becerro-De-bengoa-vallejo R Losa-Iglesias ME Rodríguez-Sanz D. Impact of the allergic asthma on foot health-related quality of life and depression: a novel case-control research. Med. (2019) 55:124. 10.3390/medicina55050124 31072062 PMC6571550

[11] Bonilla J Bernal G Santos A Santos D. A revised Spanish version of the Beck Depression Inventory: psychometric properties with a Puerto Rican sample of college students. J Clin Psychol. (2004) 60:119–30. 10.1002/jclp.10195 14692014

[12] Suárez-Mendoza AA Cardiel MH Caballero-Uribe C V Ortega-Soto HA Márquez-Marín M. Measurement of depression in Mexican patients with rheumatoid arthritis: Validity of the Beck Depression Inventory. Arthritis Rheum. (1997) 10:194–9. 10.1002/art.1790100307 9335631

[13] Vega-Dienstmaier J Coronado-Molina Ó Mazzotti G. Validez de una versión en español del Inventario de Depresión de Beck en pacientes hospitalizados de medicina general. Rev Neuropsiquiatr. (2014) 77:95. 10.20453/rnp.2014.1151

[14] Holt GR. Declaration of Helsinki-the world's document of conscience and responsibility. South Med J. (2014) 107:407. 10.14423/SMJ.0000000000000131 25010578

[15] Wilkins RA Stephensen D Siddle H Scott MJ Xiang H Horn E. Twelve-month prevalence of haemarthrosis and joint disease using the Hemophilia Joint Health score: evaluation of the UK National Hemophilia Database and Haemtrack patient reported data: an observational study. BMJ Open. (2022) 12:e052358. 10.1136/bmjopen-2021-052358 35022172 PMC8756269

[16] Beck AT Ward CH Mendelson M Mock J Erbaugh J. An inventory for measuring depression. Arch Gen Psychiatry. (1961) 4:561–71. 10.1001/archpsyc.1961.01710120031004 13688369

[17] Osorio-Guzmán M Gutiérrez-González G Bazán-Riverón GE Núñez-Villegas NN Fernández Castillo GJ. Percepción de la calidad de vida relacionada con la salud y la depresión en pacientes con hemofilia. (Spanish) Rev Medica del IMSS. (2017) 55:416–22. 28591493

[18] Kim SY Kim SW Kim JM Shin IS Baek HJ Lee HS. Impact of personality and depression on quality of life in patients with severe Hemophilia in Korea. Hemophilia. (2013) 19:1–6. 10.1111/hae.12221 23809853

[19] López López D Callejo González L Elena Losa Iglesias M Luis Saleta Canosa J Rodríguez Sanz D Calvo Lobo C. Quality of Life Impact Related to Foot Health in a Sample of Older People with Hallux Valgus. Aging Dis. (2016) 7:45. 10.14336/AD.2015.0914 26816663 PMC4723233

[20] Palomo-López P Becerro-de-Bengoa-Vallejo R Losa-Iglesias ME Rodríguez-Sanz D Calvo-Lobo C López-López D. Impact of Hallux Valgus related of quality of life in Women. Int Wound J. (2017) 14:782–5. 10.1111/iwj.12695 27928895 PMC7950007

[21] Lobo CC Marin AG Sanz DR Lopez DL Lopez PP Morales CR. Ultrasound evaluation of intrinsic plantar muscles and fascia in hallux valgus: a case-control study. Medicine (Baltimore). (2016) 95:e5243. 10.1097/MD.0000000000005243 27828846 PMC5106052

[22] Witkop ML Lambing A Nichols CD Munn JE Anderson TL Tortella BJ. Interrelationship between depression, anxiety, pain, and treatment adherence in hemophilia: results from a US cross-sectional survey. Patient Prefer Adherence. (2019) 13:1577–87. 10.2147/PPA.S212723 31571840 PMC6759218

[23] Siboni SM Mannucci PM Gringeri A Franchini M Tagliaferri A Ferretti M. Health status and quality of life of elderly persons with severe hemophilia born before the advent of modern replacement therapy. J Thromb Haemost. (2009) 7:780–6. 10.1111/j.1538-7836.2009.03318.x 19220727

[24] Cohen A Dias A Azariah F Krishna RN Sequeira M Abraham S. Aging and well-being in Goa, India: a qualitative study. Aging Ment Heal. (2018) 22:168–74. 10.1080/13607863.2016.1236239 27689842 PMC5374050

[25] Lichstein KL Scogin F Thomas SJ Dinapoli EA Dillon HR Mcfadden A. Telehealth cognitive behavior therapy for co-occurring insomnia and depression symptoms in older adults. J Clin Psychol. (2013) 69:1056–65. 10.1002/jclp.22030 24014056 PMC4563986

[26] Barcelos-Ferreira R Izbicki R Steffens DC Bottino CMC. Depressive morbidity and gender in community-dwelling Brazilian elderly: Systematic review and meta-analysis. Int Psychogeriatrics. (2010) 22:712–26. 10.1017/S1041610210000463 20478096

[27] Karakus MC Patton LC. Depression and the onset of chronic illness in older adults: A 12-year prospective study. J Behav Heal Serv Res. (2011) 38:373–82. 10.1007/s11414-011-9234-2 21293976

[28] Shapiro S Makris M. Hemophilia and ageing. Br J Haematol. (2019) 184:712–20. 10.1111/bjh.15745 30592034

[29] Wilding J Zourikian N Di Minno M Khair K Marquardt N Benson G. Obesity in the global Hemophilia population: prevalence, implications and expert opinions for weight management. Obes Rev. (2018) 19:1569–84. 10.1111/obr.12746 30188610

[30] Witkop M Neff A Buckner TW Wang M Batt K Kessler CM. Self-reported prevalence, description and management of pain in adults with Hemophilia: methods, demographics and results from the Pain, Functional Impairment, and Quality of life (P-FiQ) study. Hemophilia. (2017) 23:556–65. 10.1111/hae.13214 28419637

